# Parasite Killing in Malaria Non-Vector Mosquito *Anopheles culicifacies* Species B: Implication of Nitric Oxide Synthase Upregulation

**DOI:** 10.1371/journal.pone.0018400

**Published:** 2011-04-04

**Authors:** Sonam Vijay, Manmeet Rawat, Tridibes Adak, Rajnikant Dixit, Nutan Nanda, Harish Srivastava, Joginder K. Sharma, Godavarthi B. K. S. Prasad, Arun Sharma

**Affiliations:** 1 Protein Biochemistry and Structural Biology Laboratory, National Institute of Malaria Research (ICMR), Dwarka, New Delhi, India; 2 Vector Biology Laboratory, National Institute of Malaria Research (ICMR), Dwarka, New Delhi, India; 3 Host Parasite Interaction Group, National Institute of Malaria Research (ICMR), Dwarka, New Delhi, India; 4 Molecular Entomology Laboratory, National Institute of Malaria Research (ICMR), Dwarka, New Delhi, India; 5 Entomology Laboratory, National Institute of Malaria Research (ICMR) Field Unit, Civil Hospital, Nadiad, Gujarat, India; 6 Molecular Diagnostics Laboratory, Institute of Cytology and Preventive Oncology (ICMR), Noida, India; 7 Department of Biotechnology, Jiwaji University, Gwalior, India; Université Pierre et Marie Curie, France

## Abstract

**Background:**

*Anopheles culicifacies*, the main vector of human malaria in rural India, is a complex of five sibling species. Despite being phylogenetically related, a naturally selected subgroup species B of this sibling species complex is found to be a poor vector of malaria. We have attempted to understand the differences between vector and non-vector *Anopheles culicifacies* mosquitoes in terms of transcriptionally activated nitric oxide synthase (*AcNOS*) physiologies to elucidate the mechanism of refractoriness. Identification of the differences between genes and gene products that may impart refractory phenotype can facilitate development of novel malaria transmission blocking strategies.

***Methodology/Principal Findings*:**

We conducted a study on phylogenetically related susceptible (species A) and refractory (species B) sibling species of *An. culicifacies* mosquitoes to characterize biochemical and molecular differences in *AcNOS* gene and gene elements and their ability to inhibit oocyst growth. We demonstrate that in species B, AcNOS specific activity and nitrite/nitrates in mid-guts and haemolymph were higher as compared to species A after invasion of the mid-gut by *P. vivax* at the beginning and during the course of blood feeding. Semiquantitative RT-PCR and real time PCR data of *AcNOS* concluded that this gene is more abundantly expressed in midgut of species B than in species A and is transcriptionally upregulated post blood meals. Dietary feeding of L-NAME along with blood meals significantly inhibited midgut AcNOS activity leading to an increase in oocyst production in *An. culicifacies* species B.

***Conclusions/Significance*:**

We hypothesize that upregulation of mosquito innate cytotoxicity due to NOS in refractory strain to *Plasmodium vivax* infection may contribute to natural refractoriness in *An. culicifacies* mosquito population. This innate capacity of refractory mosquitoes could represent the ancestral function of the mosquito immune system against the parasite and could be utilized to understand the molecular basis of refractoriness in planning effective vector control strategies.

## Introduction

The mosquito *Anopheles culicifacies* Giles 1901 is the most important vector of malaria in India and is responsible for nearly 65 percent of total 2–3 million malaria cases reported annually [1]. The *An. culicifacies* species complex comprises of five sibling species provisionally designated as species A, B, C, D and E [2]. Sibling species are phylogenetically closely related to each other, are morphologically indistinguishable, and can crossbreed in captivity. Distinct biological variations are reported to exist among different members of the *An. culicifacies* sibling species complex with respect to variation in disease-transmission potential and variation in susceptibility to *Plasmodium* [2], [3], [4]: some are refractory and block transmission of parasite [5], [6]. Thus naturally evolved and genetically selected refractory strains are important for the study of mechanisms that mediate *Plasmodium* killing [7], [8], [9].

The whole genome analysis and application of genetic and molecular biological techniques to research on mosquitoes has broadened the scope for the development of disease control strategies [10], [11]. Advances in the molecular genetic manipulations of insect species have led to speculation that malaria could be controlled through genetic alterations of *Anopheline* mosquitoes rendered refractory to *Plasmodium* growth and differentiation can be used for development of novel control strategies [12], [13], [14], [15]. Among limited studies carried out so far on malaria refractory mosquito, most are restricted to animal parasite models. A specific strain of *An. gambiae* originally selected to be refractory to *P. cynomolgi* was found to have limited refractoriness to human malaria parasite *P. falciparum* [7]. Furthermore, a strain of *An. stephensi* selected for refractoriness to *P. falciparum* transmission showed no detectable resistance to other *Plasmodium* species [16]. However, none of these strains were found to be completely refractory to any of the human *Plasmodium.* While assessing natural susceptibility of *An. culicifacies sensu lato* from different geographical areas against *P*. *vivax* infection, Adak *et al* [17] reported the isolation of a naturally occurring field strain of *An. culicifacies* that is 100% refractory to *P. vivax* and partially resistant to *P. falciparum* and *P. vinckei* (rodent parasite) [6], [17]. This iso-female line has been identified as *An. culicifacies* species B and may serve as a model for the study of biochemical and molecular novel innate immune responsive strategies for mechanisms of malaria refractoriness.

To date the molecular basis of refractoriness and more generally parasite recognition and killing are not well understood. *Plasmodium* undergoes a complex sporogonic development in the midgut and salivary glands of the mosquito. During their passage through a mosquito vector, malaria parasites undergo several developmental transformations including that from a motile zygote, the ookinete, to a sessile oocyst that develops beneath the basal lamina of the midgut epithelium. This developmental cycle can be blocked by the innate cellular immune responses of the mosquito thereby resulting in the elimination of parasite in the mosquito. It has long been recognized that mosquitoes possess highly effective innate defense mechanisms of both cellular and humoral nature [18], [19], [20], [21]. Recent studies have documented a variety of additional immune responses, both cellular and humoral, and secretion and activation of antimicrobial peptides, proteins and enzymes [22] as manifested by transcriptional activation of the infection-responsive genes [15] but no specific cyto-toxic mechanism has been described for any mosquito strains. In some of the naturally selected mosquito refractory strains these responses may result in the complete blockage of parasite development. Recent studies have suggested the mosquito refractoriness to be manifested in sequential steps namely parasite recognition and parasite killing followed by melanization for disposal of dead parasites [23]. It has been shown that interference with physiological responses may affect the immune activity readout e.g PPO activating enzymes, but how these responses are coordinated and regulated is not yet known. Therefore, the drive to identify novel control strategies has focused on identifying the genes and gene products that may impart refractory phenotype manifested by immune responses for killing of parasites at developmental stages in the mosquito [20], [24].

Nitric oxide (NO), a multifunctional free radical and non specific cytotoxic antiparasitic molecule [25], [26] has been strongly suggested as an important component of innate immunity in midgut lumen. NO is also produced from the midgut epithelial cells via the oxidative deamination of L-arginine to L-citrulline which is catalyzed by nitric oxide synthase (NOS) [27]. It was shown [28] that a NOS gene in *An. stephensi* is transcriptionally activated at a modest level after malaria infection to limit the development of parasites; the early induction partly occurs in the mid-gut, but the origin of late induction has not been characterized. Induction of *AsNOS* expression is proportional to the intensity of parasite infection and is detectable in the mid-gut by 6 h post infection [29], [30], [31]. Early induction is critical to inhibition of parasite development: dietary provision of the NOS inhibitor N_-nitro-L-arginine, with a half life in blood of 3 to 6 h [29], resulted in significantly higher parasite infection intensities than did the inactive enantiomer N_-nitro-D-arginine [28]. Furthermore, basal level of NOS is required for the survival of early stage *Plasmodium,* but elevated level of NOS during later stage of oocyst development acts as major oocyst limiting factor [25]. The NO-mediated defense of *An. stephensi* [26] is analogous to mammalian NO-mediated inactivation of liver-invading sporozoites and blood-stage gametocytes [32] indicating that mosquitoes and mammals share a conserved anti-parasite defense. Interestingly, though recently other immune molecules like TEP1, APL family member proteins have been implicated for refractory mechanism [33], [34] the dynamic role of NOS in parasite killing has not been explored to examine the mechanism of refractoriness.

In the present study, we investigate the plausible mechanism of refractoriness to *P. vivax* in the malaria non vector mosquito species of *An. culicifacies* species B through NOS physiologies. Our goal is to understand and develop alternate tools for altering the vector competence of *An. culicifacies* which requires the understanding the mechanism of vectorial resistance to the malaria parasite including biochemical and molecular studies of vector parasite interactions. Interruption of transmission cycle in the mosquito by key toxic molecules namely nitrates and nitrites that are required for successful killing of the parasite in the vector would require detailed knowledge of the complex interplay between *Plasmodium* and its mosquito vector. Our research provides the first description of the NO responses of *An. culicifacies* against the human malaria parasite *P. vivax* during its interaction with the mosquito midgut and predicts the existence of *Plasmodium*-specific NOS mechanisms of gene induction in *An. culicifacies* refractory species B. We show that refractoriness may be due to cytotoxic killing of parasitic stages in the mosquito midgut lumen. Our data suggest that mosquito innate immune system may affect refractoriness via upregulation of *AcNOS* pathway and NO may contribute to killing of parasite stages. Our results demonstrated that *AcNOS* may be another effector gene in addition to prophenoloxidase pathways to block the development of the malaria parasite in *An. culicifacies* mosquito's midgut lumen and thus may elucidate a novel putative mechanism of refractoriness.

## Materials and Methods

### Study Design

The study was carried out on susceptible (Species A) and refractory (Species B) *An. culicifacies* sibling species to evaluate a plausible role of NOS gene and gene elements in biochemical and molecular terms. This study was performed in three sequential steps namely biochemical (NOS specific activity, Nitrite and Nitrate assay), oocyst kinetics (oocyst growth) and molecular (PCR, semiquantitative RT-PCR, real time PCR) at different periods of infected blood feeding.

The study was conducted under the protocol reviewed and approved by the institutional Scientific Advisory Committee (SAC) of National Institute of Malaria Research (NIMR). Written informed consent was obtained from all the volunteers prior to the collection of *P. vivax* positive blood samples collected for mosquito feeding.

### Establishment of refractory strain

The iso-female line that was identified as sibling species B and designated as *P. vivax* refractory strain was established as described by *Adak et al* [17]. Briefly, Indoor resting wild *An. culicifacies sensu lato* adult females were collected from human dwellings by hand catch method and transported to laboratory. Few adult female mosquitoes from each F_1_ iso-female progeny were identified to sibling species using species-specific diagnostic inversion genotypes as described [35]. At least 50 to 60 iso-female lines of species B from a particular geographical locality were pooled together to establish a strain. Further, progenies of a single iso-female line originated from Haldwani, Uttaranchal state, 29^o^ 23′ N, 79^o^ 30′ E was found to be 100% refractory against *P. vivax* infection were selected.

### Mosquito rearing

Cyclic colonies of Species A (S) and Species B (R) strains of malaria vector, *Anopheles culicifacies*, were reared and propagated in an insectary at National Institute of Malaria Research, Delhi as described by Adak *et al* [5]. Female mosquitoes were offered rabbit blood for ovarian development. Following hatching, larvae were reared in enamel trays containing de-chlorinated water and fed on powdered dog biscuits and brewer's yeast tablets in 3∶2 ratios.

### Blood feeding strategy

About 1–2 ml of *P. vivax* infected blood was drawn from consenting volunteer patient (aged ≥16 yrs) having mature *P. vivax* gametocytes density ranging between 0.05 to 0.5% following human use protocol approved by the Human Ethical Committee of the Centre as described by Adak et al [5]. Thin blood smears prepared from *Plasmodium vivax* positive blood were fixed in methanol and stained in JSB stain. The slides were examined under oil immersion lens of compound microscope (Carl-Zeiss, Germany) for the presence of various stages of parasite.

Three to four day old mosquitoes were starved by depriving them of raisin and glucose pads for 12–16 hours. Approximately 100–200 starved mosquitoes of both species A and species B were held in cages separately and divided into three groups namely: sugar fed (SF), uninfected blood fed (UBF) and *P. vivax* infected blood Fed (IBF). SF mosquitoes were maintained (glucose pad) under appropriate conditions and were treated as controls. For batch of UBF and IBF mosquitoes uninfected blood and gametocyte positive *P. vivax* infected blood was placed in the cage for 2 hour for feeding the female adult mosquitoes via a membrane feeding apparatus essentially following the method as described by Adak *et al.,* [5]. After 2 hour of feeding, infected blood was removed from the cage. After 30 min. unfed and partially fed mosquitoes were removed from each cohort and only fully engorged mosquitoes were kept upto 14 days securely in 30×30×30 cms cloth cages for dissection of mosquito midguts for subsequent examination of sporogonic development. Mosquitoes were maintained on glucose water soaked sterile cotton balls and changed daily, until dissection.

### Midgut and haemolymph preparation

Minimum of 50% of the surviving *An. culicifacies* mosquitoes from each feeding experiment were dissected on ice in PBS (phosphate-buffered saline). Midgut tissue samples and haemolymph samples were simultaneously isolated from individually dissected mosquitoes and were pooled (25 samples) from sugar fed (SF, 0 day) and UBF and IBF mosquitoes (1, 3, 7, 9–10, and 14–15 days). Midguts were opened by a longitudinal incision and haemolymph was directly collected and pooled. Midgut tissues were thoroughly rinsed three times in ice-cold PBS to remove all traces of peritrophic matrix and gut contents. Pooled haemolymph and dissected mid-guts tissues were sonicated on ice (three pulses for 20 sec). After sonication homogenized tissue was centrifuged at 1500 xg for 10 min to remove cell debris. Supernatant was collected and stored at −70°C for further analysis. Protein estimation was carried out by Lowry's method [36].

### Biochemical Studies

#### Inhibition on AcNOS activity

Specific activities of AcNOS in midgut lysates of SF (0 days), UBF, IBF mosquitoes in two replicates were measured as a rate of conversion of Arg to Cit at 1, 3, 7 and 9–14 days pBM using nitric oxide colorimetric assay (Roche). **T**he concentration of midgut samples were calculated from the standard curve and have been then compared by determining µmoles/μg protein/unit time.

For AcNOS inhibition experiments and to diminish the possible effects of mid-gut microflora on NOS expression and NO generation, mosquitoes were provided with 10% sugar solution and gentamicin-soaked (50 µg/ml in water) sterile cotton for two days before blood feeding. The NOS inhibitor, L-NAME (1 mg/ml) was added to the blood in one feeder, D-NAME (1 mg/ml) the inactive isomer, to the second feeder and *P. vivax* infected blood alone was added to the third feeder, as a control. Fully engorged mosquitoes were separated and transferred to cages supplied with 10% glucose solution and maintained. Midguts from 25 mosquitoes per group were dissected at different time points (1, 3, 7, and 9–14 days). Specific activities of AcNOS in midgut of infected blood fed mosquitoes with and without treatments in two replicates were measured.

### Determination of nitrite/nitrate levels

Production of NO was assessed by measuring the accumulation of nitrite/nitrate (NO_2_^-^/NO_3_^-^) in the dissected haemolymph and mid-gut from sugar fed (0 days), uninfected blood fed (UBF) and *P. vivax* infected blood fed mosquitoes (IBF) of sensitive (Sp. A) and refractory (Sp. B) species at 1, 3, 7, 9–10 and 14–15 days pBM by using a modified HPLC microassay method developed in our laboratory [37]. Statistical differences among mean absorbance of two replicates in uninfected and infected species at different days were analyzed using 2 ways ANOVA (p<0.05).

### Oocyst kinetics studies

#### Effects of NO on ookinetes viability: oocysts counts and dynamics

Two replicates of three groups of mosquitoes were provided with sugar cubes and gentamicin (50 µg/ml) in water for three days before blood feeding. On day 0, L-NAME and D-NAME was added to the infected blood before mosquito feeding through a membrane feeding device; a third aliquot was left as an untreated control. Minimum of 50% of the surviving *An. culicifacies* mosquitoes from each feeding experiment, fed on the same blood isolate were dissected on day ‘9’ and ‘10’ in normal saline (0.65% NaCl) and stained in a drop of 0.5% mercurochrome [38]. Subsequently, midguts were dissected and individual midguts were removed amd placed under a small piece of cover glass and examined for the presence of infection in the midgut under an x 10 interference phase contrast objective of Axiophot Zeiss microscope. Morphology was examined for *P. vivax* live oocysts and number of oocysts was counted.

#### Statistical analysis

Differential infection among these two strains was assessed using two outcome measures by comparing two indicators; the percent gut positivity for oocysts /encapsulated parasites (oocyst rate) and geometric mean (GM) number of oocysts/encapsulated parasites per gut (oocyst density) among all the infected mosquitoes as described earlier [17]. The oocyst prevalence was analyzed with the Chi-square Fishers exact test with Yates correction, and the oocyst density with the Kruskal-Wallis non-parametric ANOVA. Data of average number of oocysts were analyzed by Tukey's test (a>0.05 for L-NAME v D-NAME experiments).

### Molecular Studies

#### Isolation of genomic DNA and PCR analysis of *Anopheles culicifacies*

Genomic DNA (gDNA) was isolated from the midgut tissue of sensitive (Sp. A) and refractory (Sp.B) strains of mosquito by the method as described [39]. PCR assay was carried out to differentiate the sequence variations between susceptible and refractory sibling species of *An. culicifacies*. In order to test the homology of nitric oxide synthase (NOS) enzyme in various strains of *An. culicifacies* against *An. stephensi*, only those sequences were selected using BLAST, which encoded co-factors of (NOS) enzyme. We have designed primer complementary to *An. stephensi* exon 1 region (200 bp) encoding co-factor heme DOMAIN. Primers specific for those sequences were designed manually and the quality of the oligos was checked using web based software Primer 3. (http://frodo.wi.mit.edu/cgi-bin/primer3/primer3_www.cgi). Primer sequences of forward and backward region were 5′ ATGAGGACCAACTATCGGG 3′ and 5′ GCCTTGGTGACAATGCTC 3′ respectively. Selected PCR products (200 bp) amplified from genomic DNA in mosquito pools were cloned into the pUC18 at HINDIII sites and sequenced and a comparative analysis of the nucleotide sequence from both strains to others reported NOS was done using the ClustalW BLAST homology analysis. (MacVector Version 7.0).

#### Dissections and RNA extractions

The mosquitoes were dissected in a drop of ice-cold sterile DEPC-treated water and midgut from 25–30 mosquitoes was pooled in 100 µl DEPC treated PBS buffer at −70°C. The tissues were ground to homogeneity with a batterydriven hand-held homogenizer using DEPC-treated sterile grinder tips and processed for RNA extraction. Total RNA was isolated from dissected 25–30 midguts of each sugarfed (SF), uninfected blood fed (UBF) and *P. vivax* infected blood fed (IBF) mosquitoes at 0, 1, 3, 7 and 9–14 days pBM using RNeasy micro kit (Qiagen).

#### Semi-quantitative RT-PCR analysis

The relative transcript abundance and expression of NOS in naive mosquitoes (*An. culicifacies* species A and species B) was evaluated by semiquantitative RT-PCR both in uninfected (UBF) and *P. vivax* infected blood fed (IBF) mosquitoes using primers designed against *An. stephensi* at various intervals of feeding as described previously. First strand cDNA was synthesized by using oligo (dT) primer and transcriptor Reverse transcriptase (Roche) according to manufacturer protocol. *AcNOS* fragment was amplified by 35 cycles of PCR (94°C for 1 min, 50°C for 1 min and 72°C for 1.00 min) with the forward and backward primers (same as used in PCR). Transcript abundance of RNA in the midgut tissues of *An. culicifacies* species A and B were normalized to the ribosome S7 protein gene fragment and only 25 cycles were used for PCR amplification in identical environment to obtain a 200 bp amplicon. After amplification 10 µl PCR products was analyzed by 1.8% agarose gel electrophoresis. The gel image was photographed by video gel documentation system (Bio-Rad). The semiquantitative RT-PCR images were evaluated and pixels in respective bands were quantified using IPLab gel software for a 12 bit image. The results were expressed as a ratio calculated from the integrated signal of *AcNOS* amplicon bands over ribosomal protein S7 gene amplicon bands. For the mid-gut expression analysis, data from three replicates were analysed by 2 way ANOVA.

#### Real Time RT-PCR analysis

Real time RT-PCR was performed using SYBR Green RT-PCR kit (Roche Diagnostics, USA) and Light Cycler 480 system (Roche Diagnostics, USA) to measure relative transcript levels of *AcNOS* in *An. culicifacies* sp A and *An. culicifacies* sp B mosquitoes. cDNA of both the species was reverse-transcribed from 500 ng total RNA using oligo(dT) primer and transcriptor Reverse transcriptase (Roche), following the manufacturer's instructions. Assays used a total reaction volume of 20 µL incorporating 9 µl master mix with SYBR green I and 10 µM of each primer and 2 µl cDNA. Signals were normalized to the ribosome S7 protein gene fragment. The same conditions have been taken for normalizer gene S7 RNA polymerase having forward primer sequence 5' GGTGTTCGGTTCCAAGGTGA 3' and reverse primer sequence 5' GGTGGTCTGCTGGTTCTTATCC 3'. The forward and backward sequences of *AcNOS* primers for PCR are 5' ATGAGGACCAACTATCGGG 3' and 5' GCCTTGGTGACAATGCTC 3' and PCR conditions were: Initial denaturation and renaturation step were same for all primers, *i.e.*, 95°C for 5 min and 95°C for 30 sec, 50°C for 40 sec, 72°C for 30 sec of 40 cycles respectively. The fluorescence acquisition temperature was 72°C for all genes. This assay was performed thrice to minimize variations due to sample handling. Amplification specificity was further validated by melting curve analysis, generated at the end of each PCR reaction. A non-template control (NTC) was run with every assay. Normalized data were used to quantitate relative levels of a RNA in species A and species B. The threshold cycles (C_t_) were recorded for *AcNOS* and S-7 amplicons during each experiment. Difference between the C_t_ of S-7 and *AcNOS* or ΔC_t_ was determined and the relative abundance of *AcNOS* was calculated in different treatments using Comparative C_t_ method using the formula 2-ΔΔC_t_ [40].

## Results

### Effect of *P. vivax* infection on AcNOS activity

**Figure 1:**
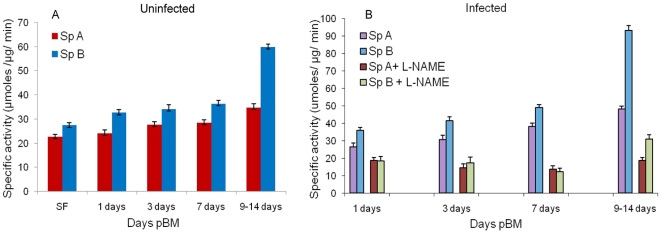
Specific activity in *P. vivax* uninfected *and* infected blood fed mosquitoes (with and without inhibitor). (A) *Anopheles culicifacies* nitric oxide synthase (AcNOS) specific activity in sugar fed (0 days) and uninfected blood fed mosquitoes midgut samples at different time points: 1, 3, 7, 9–14 days pBM in species A and species B. (B) *An. culicifacies* nitric oxide synthase (AcNOS) specific activity in *P. vivax* infected blood fed mosquitoes midgut samples at different time points: 1, 3, 7, 9–14 days pBM in species A and species B with and without L-NAME. L-NAME: N-nitro-L- arginine methyl ester, pBM:post blood meal.

In our endeavor to investigate the role of nitric oxide mediated killing of the *P. vivax* parasite in the *An. culicifacies* species B, we measured the specific activities of the nitric oxide synthase enzyme in both the sensitive (*An. culicifacies* species A) and refractory mosquitoes (*An. culicifacies* species B). We assessed the activities of AcNOS post blood meal at 1, 3, 7, 9–14 days in mosquitoes by feeding uninfected blood (Figure 1A). 0 day old sugar fed mosquito midguts were taken as a control. In these experiments, the enhanced activity of AcNOS even at day 0 in refractory species suggested that NO physiology may play a role in refractoriness. Data from blood feeding experiments showed that the AcNOS specific activity of the enzyme is increased with the day's pBM in both species A and species B. However, the increase was much more in species B as compared to species A. Compared to midgut AcNOS activitiy in *An. culicifacies* species A (34.8 µmoles/µgprotein/min, p<0.001), refractory species *An. culicifacies* species B exhibited a far more increase (60 µmoles/µgprotein/min, p<0.001) at 9–14 days pBM (Figure 1A). Thus, these data suggested that nitric oxide mechanism could contribute to the refractory phenotype of the *An. culicifacies* species B mosquitoes.

To assess the ability of *P. vivax* parasite to induce the AcNOS activity, we have also measured and compared the specific activities of the AcNOS in both the sensitive species A and refractory species B by feeding *P. vivax* infected blood meal at 1, 3, 7, 9–14 days by membrane feeding method. Data from infected blood feeding experiments showed that the AcNOS specific activity of the enzyme is rapidly increased with the days post blood meal. Compared to AcNOS activitiy in *An. culicifacies* species A (26.4 µmoles/µgprotein/min, at day 1 vs. 48.2 µmoles/µgprotein/min, at day 9–14; p<0.001), *An. culicifacies* species B exhibited a far more increase (35.9 µmoles/µgprotein/min, at day 1 vs. 93.3 µmoles/µgprotein/min, at day 9–14; p<0.001) post infected blood meal (Figure 1B).

We also tested the effect of L-NAME, a known inhibitor of nitric oxide synthase enzyme, to inhibit this AcNOS activity by feeding this inhibitor simultaneously with the infected blood meals to the mosquito's midguts. The enzyme activity was found to be markedly inhibited by this L-NAME at all days (1, 3, 7, and 9–14 days) of blood feeding in both species A and species B. The significant inhibition in the specific activity (µmoles/µgprotein/min) was however observed in the refractory species B at 9–14 days (93.3 vs. 30.9 p<0.001) (Figure 1B). Thus, these data suggested that induction of the nitric oxide synthase activity is blood and parasite induced which is inhibited by L-NAME. Higher level of NOS during *Plasmodium* infection, further indicate that refractory strains are under chronic state of hostile cyto-toxic stress because of the production of reactive cytotoxic free radicals viz NO_2_^-^, NO_3_^-^ etc. This induction of the enzyme activity in mosquito midguts of *An. culicifacies* species B may be an inheritable trait of refractory species and may lead to an increased production of reactive nitrogen species namely nitrate and nitrites which are the stable reaction products of NO and could contribute to the killing of the parasite in midguts leading to the refractory phenotype of the *An. culicifacies* species B mosquitoes.

### Effect of infection on mid-gut and haemolymph NO_2_^-^ and NO_3_^-^ levels

**Figure 2:**
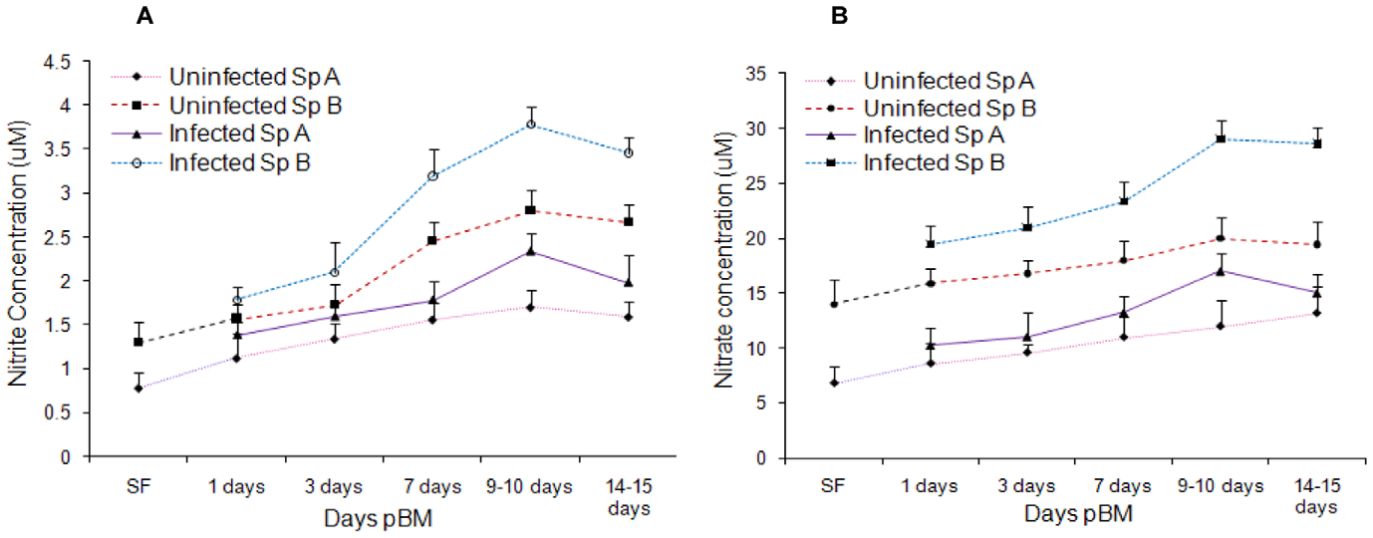
Midgut nitrite and nitrate assays. (A) Nitrite concentrations in sugar fed, uninfected blood fed and *P. vivax* infected blood fed *An. culicifacies* species A and species B midguts at 1, 3, 7, 9–10 and 14–15 days pBM. (B) Nitrate concentrations in sugar fed, uninfected blood fed and *P. vivax* infected blood fed *An. culicifacies* species A and species B midguts at 1, 3, 7, 9–10 and 14–15 days pBM. Means were analyzed by using 2 way ANOVA (p<0.05).

To investigate this possibility further, we have measured the differences in nitrate and nitrite (NO_2_^-^ and NO_3_^-^) within the *An. culicifacies* mid-gut and hemolymph during early sporogonic development of *P. vivax* under semi-natural conditions of transmission in both sensitive (Species A) and refractory species (Species B). NO_2_^-^ and NO_3_^-^ concentrations in *Anopheles* species B were found to be significantly higher than the concentrations in species A. Our data shows that mid-gut levels of NO_2_^-^ and NO_3_^-^ were significantly higher in *Plasmodium*-infected mosquitoes than in uninfected mosquitoes at all time points, with the greatest relative difference at 9 days pBM (Figure 2A and 2B). Levels at 9–10 days pBM may be correlated with induction and a higher specific activity in infected mosquitoes at this time (Figure 1B). At 14 days pBM nitrate levels in species A & B were 15.11 ± 1.73 µM and 28.6 ± 1.54 µM respectively and nitrite levels were 1.98 ± 0.32 µM and 3.48 ± 0.18 µM respectively. NO_2_^-^/NO_3_^-^ midgut concentration was appeared to be increased in infected mosquitoes at different time points (Figure 2) and same pattern was observed in AcNOS enzyme activity above, which indicates the direct correlation between these two biochemical assays.

**Figure 3:**
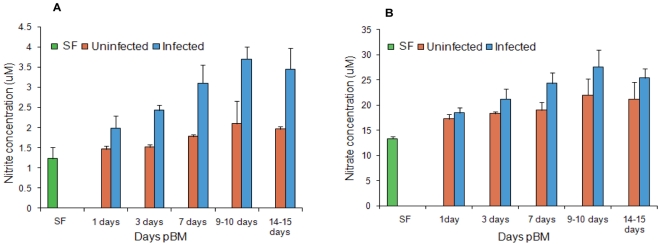
Hemolymph nitrite and nitrate assays. (A) Nitrite concentrations in sugar fed, uninfected blood fed and *P. vivax* infected blood fed *An. culicifacies* species B hemolymph at 1, 3, 7, 9–10 and 14–15 days pBM. (B) Nitrate concentrations in sugar fed, uninfected blood fed and *P. vivax* infected blood fed *An. culicifacies* species B hemolymph at 1, 3, 7, 9–10 and 14–15 days pBM. Means were analyzed by using 2 way ANOVA (p<0.05).

We have also determined the hemolymph NO_2_^-^ and NO_3_^-^ concentration in SF (0 day), UBF and IBF *An*. *culicifacies* B at different time periods (1, 3, 7, 9–10 and 14–15 days). Hemolymph NO_2_^-^ and NO_3_^-^ concentration of blood fed *P. vivax* infected species B were found to be higher than uninfected mosquitoes This concentration of NO_2_^-^ and NO_3_^-^ were found to be time dependent and were proportionately higher in *Plasmodium* infected mosquitoes than in uninfected mosquitoes at all time points with the greatest relative difference at 9–10 days pBM (Figure 3A and 3B).

### Oocyst kinetics

We tested the ability of *An. culicifacies* species B to support development of *P. vivax*. *An. culicifacies* species A mosquitoes were used as a reference. Three to four-day-old female mosquitoes were fed via a membrane with gametocytes infected *P. vivax* and 9-10 days later their midguts were dissected and the presence of oocyst and the total parasites per midgut were recorded. The results from two independent feeding experiments showed 0 of 22 (0/22) and 7 of 39 (7/39) midguts infected (0% and 17.9% oocyst infection prevalence, respectively) in *An. culicifacies* species B and the corresponding median oocyst densities were 0.0 and 2.0 in both experiments (Table 1). In the paired feedings of *An. culicifacies* species A 16/48 and 14/34 midguts had at least some viable oocyst (33.3% and 41.1% oocyst infection prevalence, respectively) with corresponding median oocyst densities of 9.0 and 12.0. The live oocyst density was found to be much lower (P<0.001) in *An. culicifacies* species B than in *An. culicifacies* species A. The distributions of oocyst densities varied significantly (P<0.01) between the two mosquito species, as one-third of *An. culicifacies* species B were observed to have virtually no oocysts whereas almost every *An. culicifacies* species A midgut had one or more (Table 1). Clearance of pre-oocyst parasitic stages and melanization of ookinetes are established important immune reactions of mosquitoes against *Plasmodium* indicating that the inherent mosquito immunity may be contributing to the reduced susceptibility of *An. culicifacies* species B.

**Table 1:** Oocyst prevalence and *Plasmodium vivax* infection density in *An. culicifacies* species A and species B.

Experiment.	Species	n	Oocyst prevalence (%)	P	Oocyst density	Range	P
1	*An. culicifacies* species A	48	27.08 (13/48)	-	9.0	1–43	-
	*An. culicifacies* species B	22	0 (0/22)	ns	0.0	0	<0.01
2	*An. culicifacies* species A	34	41.17 (14/34)	-	12.0	1–48	-
	*An. culicifacies* species B	39	17.94 (7/39)	<0.001	2	1–5	<0.01

Mosquito mid-guts were examined for *P. vivax* live oocysts 9–14 days post infection. Oocyst prevalence is the percentage of mosquitoes displaying at least one live oocyst, and Oocyst density is the median number of oocysts in infected mosquitoes infection of *An. culicifacies* species B was not observed in experiment 1. The oocyst prevalence was analyzed with the chi-square Fishers exact test with Yates correction, and the oocyst density with the Kruskal –Wallis non-parametric ANOVA. n: number of mosquitoes, ns: not significant.

**Figure 4:**
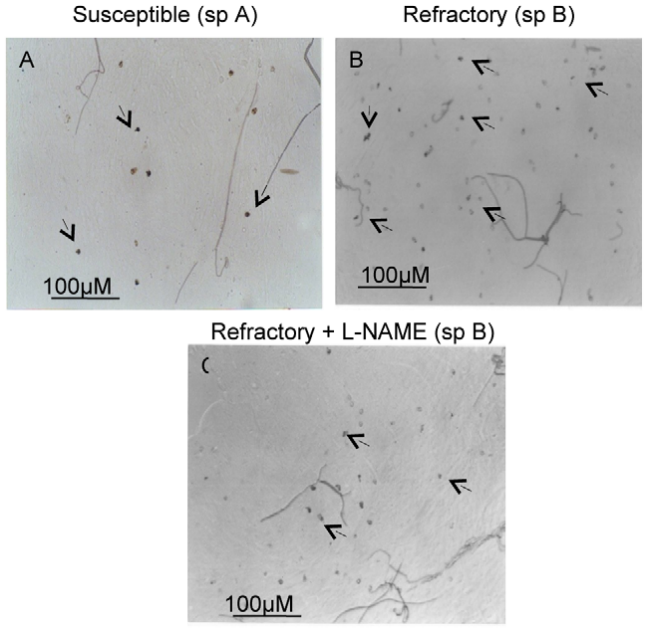
*Plasmodium* parasite killing in *An. culicifacies* (species A and species B). (A) Melanized ookinetes (arrows) of *P. vivax* in sensitive *An. culicifacies* species A. (B) Melanized ookinetes (arrows) of *P. vivax* in refractory *An. culicifacies* species B while crossing the *An. culicifacies* midgut. (C) Melanized ookinete in the midguts of *An. culicifacies* species B (refractory) infected with *P. vivax* treated with NOS inhibitor L-NAME. Four independent paired experiments were performed. Treatment with L-NAME decreased the melanized ookinetes count (C) in comparison to without treatment (B).

**Figure 5:**
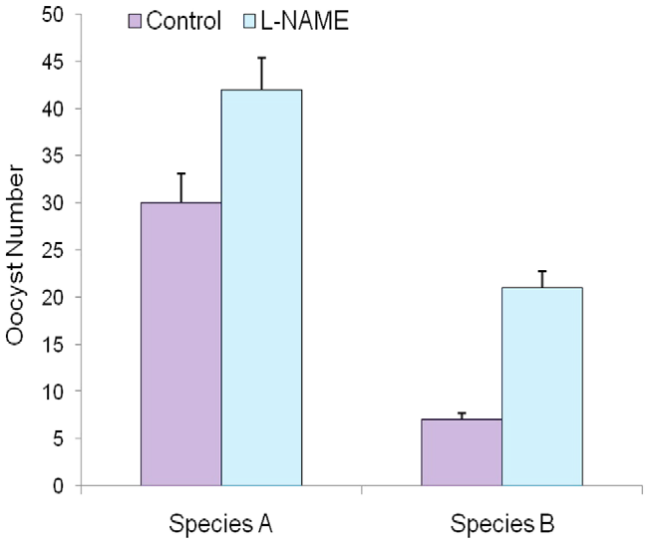
Live oocyst density in the midguts of *An. culicifacies* species A and species B infected with *P. vivax*. The geometric means ± SD of the pooled data from the two independent experiments are shown (Biological replicates). The live oocyst densities (purple bars) were 30±3.4 for *An. culicifacies* species A (n = 47) and 7±0.5 for *An. culicifacies* species B (blue bars) (n = 22; P<0.001), and the oocyst densities were 42 ±2.9 and 21±1.7, respectively (P<0.001) following the L-NAME treatments (n: number of midguts). Oocyst mortality increased with the L-NAME treatment in both species A and species B.

To support this conclusion, we examined the effect of L-NAME, which appeared to interfere with induced *AcNOS* (Figure 1B) on *P. vivax* development in mosquitoes. No much difference in survivorship was observed across the treatments in species A. We assessed the mosquito immunity in terms of NOS physiology by measuring the effect of NOS inhibitor L-NAME on the melanized ookinetee density in refractory species to determine whether this is correlated to the killing of the oocysts in the midguts of the refractory species B (Figure 4). We observed that the melanized ookinetes were reduced when the mosquitoes were fed with L-NAME (Figure 4). We however, did not count the number of melanized ookinetes. Simultaneously, we also observed that when the refractory mosquitoes *An. culicifacies* species B were subjected to L-NAME treatments the live oocyst count increased in the mosquito midguts (Table 2). This increase was substantial (7 verses 21) in species B as compared to species A (30 verses 42) (Figure 5). However, when the mosquitoes were fed with D-NAME (Table 2) there was not much effect on the parasite densities of species A (30 verses 39) or in species B (7 verses 9). These data therefore may suggested that nitric oxide mediated the cytotoxic effect on the parasites in the midguts of species B leading to increased melanization of dead ookinetes which was reduced with the feeding of L-NAME (Figure 4). Inhibition of *AcNOS* by L-NAME leads to a reduction of nitrate and nitrites in the midguts. This could contribute to the reduced susceptibility of *An. culicifacies* species B which leads to increase in live oocyst count.

**Table 2:** Effect of L-NAME and D-NAME treatment on oocyst count in *Anopheles culicifacies* species A and species B.

Experiments	Oocyst number/Midgut		
	Sugar + Gentamicin (50 µg/ml)	L-NAME (1 mg/ml)	D-NAME (1 mg/ml)
*An. culicifacies* species A	30 ± 3.4 (47)	42 ± 2.9 (49)	39 ± 3.8 (50)
*An. culicifacies* species B	7 ± 0.5^a^ (22)	21 ± 1.7^b^ (26)	9 ± 0.6^a^ (25)

*P. vivax* infected blood fed mosquitoes were maintained on sugar and L-NAME or D-NAME treated or untreated water until dissection at 7 days pBM to count midgut oocysts. Figures in the table are depicted as average no. of oocysts per gut ± SEM. Values in parentheses depict the total no. of midguts dissected. Data were analyzed by Tukey's test (a>0.05 for L-NAME v D-NAME experiments, b<0.05 for L-NAME v D-NAME experiments). Significant differences are indicated by different letters. pBM: post blood meal, NAME: N_w_-nitro-arginine methyl ester, SEM: standard error mean.

### Sequence homology and Phylogenetic analysis of *AcNOS* gene

Since we have observed that the AcNOS activity, nitrate and nitrite levels are higher in the *An. culicifacies* species B at all times before and during the blood feeding experiments, we therefore speculate that the refractory mechanisms of *An. culicifacies* Species B in terms of nitric oxide physiologies may be under genetic control. We have therefore used a PCR-based strategy to amplify exon sequences of *AcNOS* gene from a genomic DNA pool constructed from midguts tissue of *An. culicifacies* species A and species B adult females using a pair of primers which were designed from the conserved cofactor-binding domain of NOS sequences from *Anopheles stephensi.*

**Figure 6:**
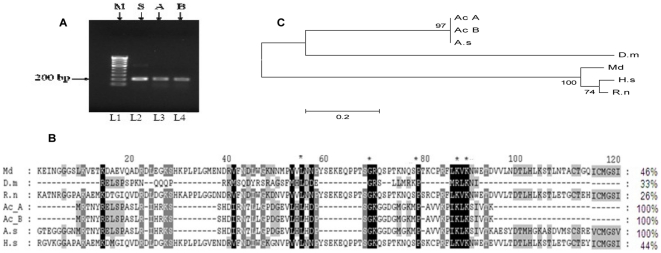
Homology analysis of *An. culicifacies* sp A and sp B. (A) PCR amplification of Exon-1 region (200 bp) fragment of mosquito nitric oxide synthase (NOS): L1: 100 bp marker (M); L2: *An stephensi* (S): L3: *An. culicifacies* sp A: L4: *An. culicifacies* sp B. (B) Clustal alignment of AcNOS with known homologus NOS sequence of other insect and vertebrate. Highly conserved and similar residues have been shown dark black & grey respectively. Relative sequences identity has also been shown. (C) Phylogenetic bootstrap consensus tree based on amino acid sequence alignment using Neighbor- joining method. Length of Horizontal lines is proportional to the minimum number of amino acids differences required to join nodes. Numerical numbers in the nodes are bootstrap confidence intervals which were calculated by 1000 heuristic search replicates. The evolutionary distances were computed using the Poisson correction method. Species name and respective sequences accession numbers includes; M. d: *Monodelphis domestic* (XP_001362705.1); D. m: *Drosophila melanogaster* (AAF25682.1); R.n: *Rattus norvegicus* (NP_434686.1); A.c_A: *Anopheles culicifacies* sp A (FJ172997); A.c_B: *Anopheles culicifacies* sp B (FJ172998); A.s: *Anopheles stephensi* (061608.2); H.s: *Homo sapiens*(NP_000611.1).

Our data on sequencing and ClustalW alignment of the amplified fragments revealed a high degree of sequence similarity (100%) between *An. culicifacies* sp.A and sp.B for *AcNOS* genes (accession number: FJ172998 and FJ172999 respectively) (Figure 6). The amplified sequence encodes a 200 residue region (Figure 6A) which revealed 33–100% identical at the amino acid level to the corresponding region of these known NOS sequences, as well as 100% identical to the recently isolated *An. stephensi* NOS sequence (Figure 6B). A phylogenetic tree (Figure 6C) was constructed on the basis of alignment of the partial *AcNOS* amino acid sequence and the corresponding homologous regions of several invertebrate and vertebrate NOS. Hence in the resulting dendrogram *An. culicifacies* species A and species B were in same clade and vertebrate NOS were in the different clade i.e. obtained by the Neighbor-joining method (Figure 6C). The deduced amino acid sequences of *Monodelphis domestic* NOS, *Drosophila melanogaster* NOS, *Rattus norvegicus* NOS, *Homo sapiens* NOS, *Anopheles stephensi* NOS and *Anopheles culicifacies* species A and species B NOS show the highest level of homology to vertebrate neuronal NOS, followed by decreasing homologies to vertebrate endothelial and inducible NOS genes.

### Differential expression of Nitric oxide synthase (*AcNOS*)

**Figure 7:**
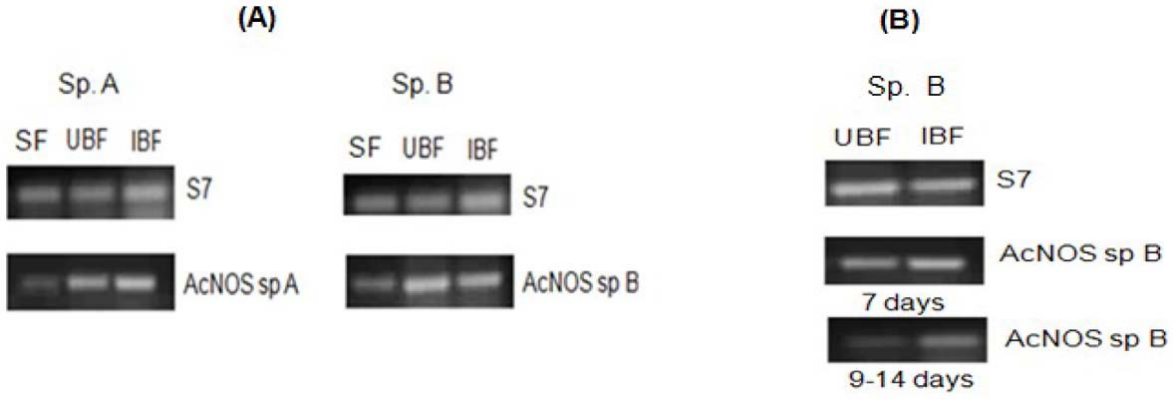
Agarose gel showing semi-quantitative RT-PCR assayed transcriptional induction of *AcNOS* in parasite induced midguts. (A) Transcriptional induction in SF, UBF, IBF *An. culicifacies* species A and species B (24 hr pBM) (B) Transcriptional induction of *An. culicifacies* species B (UBF, IBF) on 7 days and 9–14 days pBM. All cDNA templates were normalized for equal yield of ribosomal protein S7 RT-PCR products. SF: sugar fed, UBF: uninfected blood fed, IBF: infected blood fed.

Since it is not possible to correlate *An. culicifacies* species A and *An. culicifacies* species B using PCR as both the species were found to be identical, the relative expression level of *AcNOS* transcript in both *P. vivax* infected sibling species was analyzed by using primers designed against *An. stephensi* phenotypes by semi-quantitative RT-PCR (Figure 7A). First we measured the basal level of NOS expression pattern by semi-quantitative RT-PCR in the midgut tissue collected at 24 hr post feeding using constitutively expressing S7 Ribosome protein gene as an internal control. As expected, in comparison to species A, we observed an increased NOS expression observed in *An. culicifacies* species B (Figure 7A).

**Figure 8:**
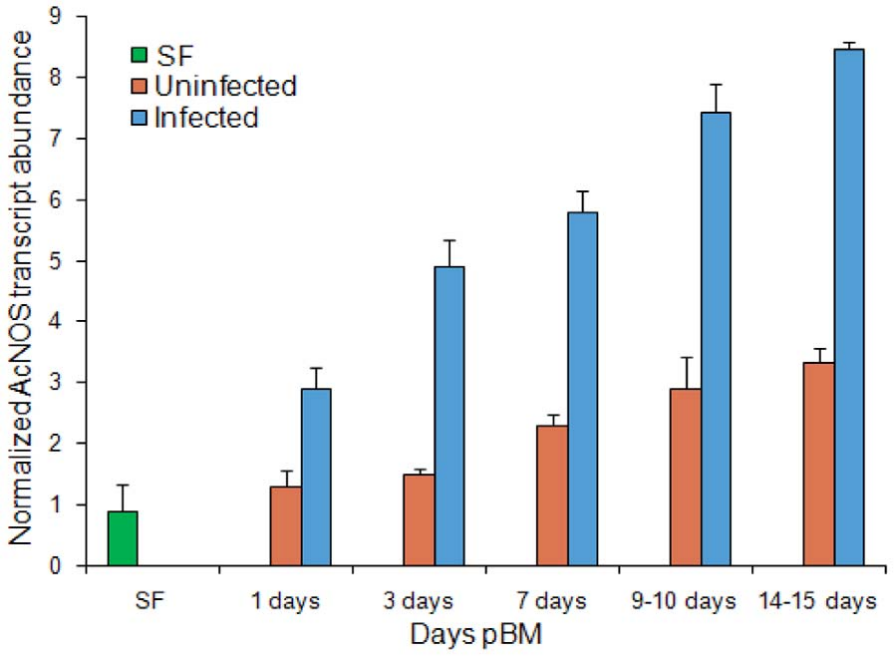
Transcript abundance in sugar fed, uninfected and *Plasmodium vivax* infected *An. culicifacies s*pecies B midguts. Total RNA from midguts of sugar fed (SF), uninfected and *P. vivax* infected blood fed was assayed using semi-quantitative RT-PCR for *AcNOS* at 1, 3, 7, 9–10 and 14–15 days pBM. The RT-PCR images were evaluated and integrated pixel values in respective bands were quantified on a grey scale and normalized to the ribosome S7 protein transcripts. Data with in each treatment from 3 replicates (biological replicates) were analyzed using 2 way ANOVA.

NOS induction was seen at all stages of parasite development in both species A and species B. Interestingly, NOS expression was found to be much more in *Anopheles species* B at 7 days in comparison to the 9–14 days (Figure 7B). At 9–14 days pBM, however, when *AcNOS* expression was induced in infected mosquitoes, the specific activity was 2-fold higher in infected mosquitoes. Increase levels of NOS expression in mid-guts of species B mosquitoes fed on *P. vivax* infected blood containing parasites and gametocytes may reveal that early sporogonic stages of *P. vivax* are able to increase NOS expression from day 1 to day 14. *AcNOS* was expressed constitutively and was transcriptionally upregulated post blood meals in the refractory species B. A proportional increase in transcript abundance of 12 fold from day 1 to day 14 was observed (Figure 8) through out the blood feeding experiments in *P. vivax* infected mosquitoes species B.

**Figure 9:**
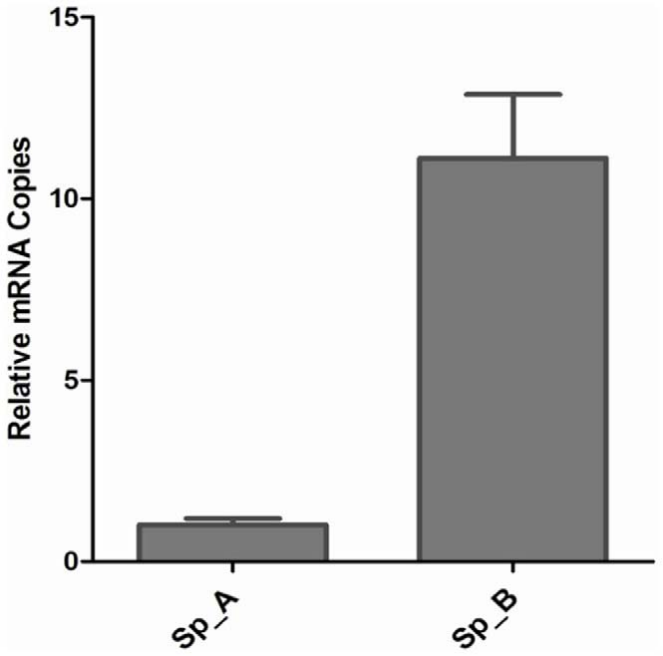
Expression pattern of NOS in mid gut of *An. culicifacies S*p A and Sp B (n = 25–30*)* at 7 days pBM. Relative transcript abundance of AcNOS in *P. vivax* blood infected susceptible (species A) and refractory strain (species B). Gene transcript quantity was measured by relative RT-PCR using the internal standard S7 RNA polymerase gene. Error bars represent standard deviation from three independent experiments (PCR replicates) are shown.

Finally for a comparative study of the relative abundance of the NOS transcripts in *An*. *culicifacies* susceptible and refractory species, a real-time RT-PCR analysis was undertaken on samples from midgut tissues at 7 days pBM. Temporal expression of *AcNOS* was monitored by real time PCR after feeding of mosquitoes (Figure 9). The threshold values (Ct) show that NOS transcript in midgut tissue was 21.13 for *An. culicifacies* species A and 18.56 for *An. culicifacies* species B. The lower Ct value of NOS for species B indicates that NOS is more abundant in the midgut tissue of species B. Thus, in *An. culicifacies*, unexpectedly we observed 11 fold induction of NOS gene in refractory species as compared to susceptible species.

## Discussion

The *Plasmodium* undergoes a complex developmental interplay during its lifecycle in mosquitoes. This interaction between vector and parasite is essential for malaria transmission. The capacity of mosquitoes to transmit malaria is determined by numerous factors such as their longevity, feeding preference, and permissiveness to parasite development in the mosquito midgut [41]. It is clear that species specific mosquito-parasite strain combinations are essential for the development of parasite in the mosquitoes. To this end, not all mosquito-parasite strain combinations are compatible: some *Plasmodium* strains are unable to develop in certain refractory mosquito strains because of innate immune system in mosquitoes that eliminate all ingested *Plasmodia* [12], [16], [22].

Most of the recent research efforts have focused mainly on blocking parasite development in mosquitoes by targeting gametocytes [32], ookinettes [42] in the mid guts of the mosquitoes. Research concerning mid gut biology exploits parasite entry mechanisms [43], melanization [44], and transcriptionally activated mosquitoes immune response related antiplosmodial genes [45] and free radicals namely ROS, RNS and nitric oxide [46], [47]. Advances in molecular biology have allowed us to characterize mosquitoes that reduce *Plasmodium* transmission [14]. The development of genetically modified *Anopheles* mosquitoes and transformation and availability of mosquitoes that exhibit enhanced refractoriness to *Plasmodium* spp. is regarded as a model for potential strategies for control of malaria transmission [48], [49].

### Refractory species

Naturally occurring, malaria resistant strains are termed as refractory mosquitoes and these are very rare in the field. Low occurrence of such resistant phenotypes is attributed to acute selection pressures on the innate immune system of mosquitoes. *Anopheles culicifaces*, a rural Indian vector of malaria is a complex of five sibling species, of which species B has been known to be a refractory species [17]. Sibling species are phylogenetically closely related to each other, are morphologically indistinguishable, and can crossbreed in captivity; however, they vary greatly in their capacity to transmit human malaria. The isolation of a wild *An. culicifaces* strain B which is refractory to *Plasmodium vivax* infection [6], [17] presents an opportunity to use this laboratory model of infection for research related to understanding the molecular basis of refractoriness.

### Induction of parasite killing

Malaria parasites are often killed by various intracellular events that may include nutrient deprivation, lysis or melanization, drug treatments and the presence of nitric oxide (NO) and reactive oxygen species (ROS) and nitrogen species (RNS) in the mosquito midgut epithelium, which are controlled by reactions of the mosquito innate immune system leading to refractoriness in mosquitoes. Large losses in parasite numbers occurs at the ookinete-to-oocyst transition stage of its life cycle [50], [51]. These losses are correlated with transcriptional activation of innate immunity genes by malaria infection during invasion of epithelial tissues and translocation to the salivary glands [52]. Three molecules with recognition functions, TEP1, LR1M1 and APL1 also have parasite killing activity however, the mechanism is incompletely understood [33], [34], [53]. The search for antiparasite effectors in the refractory species *Anopheles* has identified some promising targets in the immune-responsive mosquito, including melanotic encapsulation [44], [52], [54], antimicrobial peptides such as defensins [55], nitric oxide synthase [56]. NO or its derivatives, play a role in the immunological reaction of the host defense against the parasites [57]. NO has also been known to produce nitrosative stress which may lead to apoptosis by activation of mitochondrial apoptotic pathway [58]. These toxic molecules may thus act triggers of apoptosis and elimination in *Plasmodium* killing in refractory mosquitoes. The availability of NO through white blood cells, through blood feeding and through mid gut cells has indisputable effects for gametocyte and ookinete killing in the mosquito midguts [32], [42].

### Implication of nitric oxide in *Plasmodium* killing in refractory species

The enzyme NOS is responsible for the formation of nitric oxide (NO) which, is involved in many physiological processes such as vasodilator activity in saliva [59], neurotransmission in brain [60] and defense killing of bacteria and macroparasites. Previous studies on vertebrate and invertebrates has shown that, among other physiological functions, nitric oxide is universally involved in immune responses, acting as signaling as well as cytotoxic molecule [26]. In this study we explored the possible role of NO in refractory mechanism *in vivo* on phylogenetically related susceptible (species A) and refractory (species B) sibling species of *An. culicifacies* in terms of nitric oxide physiologies and NOS mediated innate immunity. Here, we have shown that AcNOS exhibit potent antagonistic effects against *Plasmodium* species via the production of reactive nitrogen intermediates in sensitive and refractory sibling species. These reactive nitrogen species are implicated in the killing of the parasites as reported previously [46], [47], [56]. This observation strongly supports the previous observation that NOS is a dynamic multifunctional enzyme that not only required for physiological integrity, but also participate as an important effectors molecules of innate immunity.

We extended our studies to examine the possible involvement of AcNOS in refractoriness of mosquito *An. culcifacies.* Interestingly, compared to susceptible strain of *An. culicifacies* species A, refractory species B exhibited an increased AcNOS enzyme activity, nitrates and nitrites in midguts and hemolymph. The similarity of *AcNOS* induction patterns following feeding on uninfected blood and infected blood suggests that parasite infected blood acts as an important signal for *AcNOS* induction prior to 1 day of infection. In the current study, elevated levels of NO synthase (NOS), an enzyme critical for the production of NO, were noted in the midguts of *P. vivax*-infected *An. culicifacies* on 7 days post infection compared with control mosquitoes. AcNOS activity in infected blood fed mosquitoes of species A was relatively changed only slightly by L-NAME at 7 days, whereas activity in infected blood fed mosquitoes in species B was significantly inhibited by L-NAME (*P*<0.005, Figure 1B). Although the production of NO may be induced by the presence of the parasite in the mosquito, this production also could be due to tissue damage or stress created by the invading parasite where higher level of toxic byproducts may involved in the parasite killing by unknown mechanism. However, regardless of the NO induction trigger, the parasite burdens in the mosquito, although not eliminated, seem to be negatively affected by NO production [25].

### Effect of L-NAME on Oocyst development

The malaria parasite is at its most vulnerable stage within the mosquito midgut, less than 10% ookinetes successfully cross the midgut epithelium and form oocysts. After this, the number of parasites dramatically increases when each oocyst generates several thousand sporozoites [61]. Thus, the strong bottleneck in parasite numbers makes ookinetes, an ideal target for interference with transmission. We tested the effect of NOS on the oocyst development of human malaria parasite *P. vivax*. Refractory species, *Anopheles culicifacies* species B exhibited a reduced oocyst prevelence in their midguts 9–10 days post infection as compared to susceptible species. However, we have also observed a significant increase in the live oocyst density in the refractory strain when fed on infected blood meal supplemented with L-NAME. The effects of L-NAME suggest that parasites are targeted before or during the oocyst development, a hypothesis which seems to be consistent with the demonstrated susceptibility of parasites to NO damage [56]. NOS-specific inhibitor specifically fully rescued the susceptibility phenotype in refractory mosquitoes, causing an approximate 4-fold increase in the oocyst density, and 71% increase in oocyst prevalence. The increase in the number of oocysts observed in response to infected blood in *An. culicifacies* species A could be an amplified midgut response to parasite infection: however, this midgut response seems to be significant in *An. culicifacies* species B. Furthermore, feeding of NOS inhibitor L-NAME, increases mean oocyst infections while D-NAME, the inactive isomer has no effect (Table 2). Luckhart et al [28] has reported similar effect when *An. stephensi* mosquitoes were fed on L-NAME and D-NAME. A decreased oocyst infection in *An. culicifacies* species B (Table 2) may be attributed to the susceptibility of *P. vivax to Anopheles culicifacies* midgut stages to nitric oxide (NO). These data clearly demonstrated that *AcNOS* could be an important player in the development of refractory nature of the mosquito and need to be explored further.

### Transcriptional upregulation of *AcNOS* gene

Our knowledge of NOS function in invertebrates is still limited [62], and the gene has only been cloned from a few insect species [28], [60]. In view of the multiple physiological roles of NO, it is quite possible that the effects on NOS will prove to be related to the success of parasite infection [28]. Our sequence analysis results of the amplified fragment of *AcNOS* gene revealed a high degree of sequence similarity between *An. culicifacies* species A and species B. This was not surprising as the two species are very closely related in the evolutionary scale and genetic introgression has likely taken place for some time after their separation and may be regarded more as a reflection of gene ancestry than functional activity [54].

To evaluate the temporal expression of *AcNOS* gene in *An. culicifaces* species A and species B experiments were designed to determine whether *P. vivax* parasite could transcriptionally induce the upregulation of NOS gene by semiquantitative RT-PCR and real time PCR analysis. Our data have concluded that *AcNOS* gene is more abundantly expressed in midgut of species B than in species A. We show that NOS expression is transcriptionally upregulated in the midgut in response to *Plasmodium* infection and induction of NOS is proportional to the intensity of infection [63]. Our results show that *An. culicifaces* does have a shared evolutionary history with *P. vivax* and thus in principle host resistance mechanisms, including *AcNOS*, could have been selected by the parasite, although to date this have not been proven. We hypothesize that upregulation of mosquito innate cytotoxicity due to NOS in refractory strain to *Plasmodium vivax* infection may contribute to natural refractoriness in *An. culicifacies* mosquito population. This innate capacity of refractory mosquitoes could represent the ancestral function of the mosquito immune system against the parasite and could be utilized to understand the molecular basis of refractoriness in planning effective vector control strategies. A better understanding of the natural mechanisms of host defense against the *Plasmodium* parasite may provide new targets for therapeutic intervention in this disease.

### Conclusions

Attention has largely focused on mosquitoes innate immune responses that may lead to cytotoxic killing, lysis and melanization of the parasites. NO, RNS and ROS are known to kill parasites in oxidation-reduction reactions and may play a role in the refractory mechanisms. However, the importance of NOS activity in inducing cytotoxic killing of the parasites needs to be ascertained in order to show the feasibility to augment the activity of NOS for killing of the parasites. In this study, we have utilized a naturally selected non-vector *An. culicifacies* species B in conjunction with the *An. culicifacies* species A as a model vector system, to understand the differences contributing to its reduced vectorial capacity. The phenotype of this refractory *An. culicifacies* species B strain is identical to that of other *An. culicifacies* sibling species complex. Dissecting the molecular basis of refractoriness in *Anopheles culicifacies* model system may pave the way to novel disease control mechanisms. Experimental evidences relating to increased NOS activity and reduced oocyst development do suggest the evolutionary significance of the existence of a cytotoxic innate immunity system operating in refractory species. It is therefore, tempting to speculate that persistent interaction of *An. culicifacies* with *P. vivax* might have led to an evolutionary co adaptation between the mosquito immune responses and this parasite, whereas the refractory phenotype could represent the ancestral function of the mosquito immune system against the parasite. This immune system could operate via triggering of mosquito AcNOS activity by the *Plasmodium vivax* dependent manner. The latter implying that AcNOS cytotoxic mechanism of parasite killing in the mosquito midgut lumen could be an important transmission blocking strategy. Based on the present data on *Anopheles culicifacies* NOS which has been shown to be transcriptionally regulated, we believe that the study of this gene is a promising approach to unravel yet unknown NOS-dependent production of nitric oxide elements, leading to a better insight in different aspects of insect physiology in terms of refractoriness. Future research will aim to determine if any of these changes or any cofactors to the enzyme AcNOS can enhance or otherwise alter the function of this *AcNOS* gene and gene elements, thus contributing to the refractoriness phenotype for novel malaria control strategies.
